# From Felbamate to Carbamazepine: Clinical Consequences of Unintentional Antiepileptic Substitution in Refractory Epilepsy

**DOI:** 10.7759/cureus.88362

**Published:** 2025-07-20

**Authors:** Natanael Duarte, Omarlyn Ruiz, Elidenia Velásquez

**Affiliations:** 1 General Medicine, Centro Materno Infantil del Nordeste, San Francisco de Macoris, DOM; 2 School of Medicine, Universidad Católica Nordestana, San Francisco de Macoris, DOM; 3 Neurology, Centro Materno Infantil del Nordeste, San Francisco de Macoris, DOM; 4 Neurology, Universidad Católica Nordestana, San Francisco de Macoris, DOM

**Keywords:** carbamazepine, case report, felbamate, lennox-gastaut syndrome, refractory epilepsy

## Abstract

Epilepsy is the most common chronic neurological disorder and one of the leading causes of disability. We report a rare and clinically significant complication involving the inadvertent substitution of felbamate with carbamazepine in a 37-year-old man with refractory epilepsy. He developed drowsiness, ataxia, dysarthria, and vomiting, followed by hematological abnormalities and respiratory symptoms. Although carbamazepine levels were within the reference range, a probable diagnosis of acute carbamazepine toxicity was made in the setting of refractory epilepsy. Therapeutic interventions included discontinuation of carbamazepine, antimicrobial therapy, and supportive care. Inadvertent substitution of antiepileptic medications in patients with refractory epilepsy can lead to serious multisystem complications. Vigilant medication management and multidisciplinary care are essential to prevent adverse outcomes.

## Introduction

Epilepsy is the most prevalent chronic neurological condition and a leading cause of disability [1]. It is characterized by repeated, unprovoked seizures that can be either generalized or focal [1]. Although numerous antiseizure medications (ASMs) are available, approximately 30% of individuals with epilepsy still experience seizures despite treatment, a condition known as drug-resistant epilepsy (DRE) [2]. Lennox-Gastaut syndrome (LGS), a severe type of epilepsy that begins in childhood, highlights this difficulty, as it often requires the use of rarer antiepileptic medications like felbamate, an option typically reserved for refractory cases because of its potential for significant side effects [3,4].

In the context of refractory epilepsy, maintaining strict adherence to the prescribed antiepileptic drug regimen is vital for effective seizure control [5]. Even minor changes in dosage due to medication substitution can lead to severe neurological and systemic complications, potentially resulting in hospitalization or, in extreme cases, death [6].

Frequently used ASMs such as phenytoin, carbamazepine, valproate, lamotrigine, and levetiracetam are generally selected according to factors such as seizure type, epilepsy syndrome, patient age, and overall health status [5]. Although these medications are effective for many individuals, their limitations can pose challenges in achieving optimal control of epilepsy [5].

We present the case of a 37-year-old man with refractory epilepsy who experienced acute neurological and systemic deterioration following the inadvertent substitution of felbamate with carbamazepine. Although carbamazepine serum levels were within the therapeutic range, the patient exhibited signs consistent with carbamazepine-related neurotoxicity. This paradox may be explained by individual sensitivity, absence of prior exposure, or the abrupt initiation of a full-dose regimen without gradual titration. Additionally, patients with underlying cerebral atrophy or chronic epilepsy may be more vulnerable to the central nervous system effects of antiepileptic drugs, even at therapeutic concentrations. This case highlights the clinical consequences of antiepileptic drug mismanagement, the importance of precise pharmacologic continuity, and the role of multidisciplinary care in addressing complex drug-induced complications.

## Case presentation

A 37-year-old single, unemployed male from Connecticut, United States, presented to the emergency department of the Centro Médico Materno Infantil del Nordeste in San Francisco de Macorís, Dominican Republic, on January 26, 2025, with a two-day history of progressive drowsiness, generalized weakness, epigastric pain, ataxia, and three episodes of non-bilious, non-bloody vomiting containing food particles. The patient’s mother reported that his regular antiepileptic medication, Felbamate 400 mg, had run out three days earlier. In an attempt to replace the medication, she obtained Tegretol (Carbamazepine) extended-release 400 mg from a local pharmacy, mistakenly believing it to be an equivalent substitute. The patient has a documented history of epilepsy requiring multiple antiepileptic medications over time, with limited response, supporting the diagnosis of poorly controlled DRE. Based on this history, recent medication changes, and clinical presentation, he was admitted with working diagnoses of DRE, suspected acute carbamazepine intoxication, and moderate dehydration.

The patient had a known history of epilepsy since the age of 8. He denied any known drug allergies, previous blood transfusions, or other neurological diseases apart from epilepsy. He reported multiple falls from standing height, likely due to seizure activity. His chronic medications included divalproex sodium 500 mg (2.5 tablets daily), escitalopram 20 mg (once daily), clobazam 10 mg (2.5 tablets daily), brivaracetam 100 mg (twice daily), and felbamate 400 mg (5.5 tablets daily). Surgical history was notable for bilateral eye fenestration of unknown indication. He reported occasional caffeine use and had a family history of maternal diabetes mellitus and paternal hypertension and dyslipidemia.

Upon arrival, the patient was somnolent but arousable to painful stimuli, with a Glasgow Coma Scale (GCS) score of 11 (O2, V4, M5). He was disoriented to place and time. Cranial nerve examination was unremarkable. Motor examination revealed globally decreased muscle strength (4/5), preserved tone, and intact deep tendon reflexes. No sensory deficits were noted. Gait evaluation revealed a broad-based, ataxic pattern with irregular steps, truncal oscillations, and lateral deviation to the left. Dysmetria was observed on finger-to-nose testing. Romberg sign was negative. Mild dysarthria and spontaneous horizontal nystagmus were present. There were no signs of meningeal irritation. Babinski and Hoffman signs were both absent.

The patient was normocephalic with isochoric and photoreactive pupils. Cardiopulmonary examination revealed a hyperdynamic precordium without murmurs. Lung auscultation revealed diminished breath sounds, scattered rhonchi, and crackles in the left lung field. Abdominal examination showed a distended but soft and depressible abdomen, with tenderness to superficial palpation in the epigastric region. No organomegaly was noted. The extremities were symmetrical and without edema.

Initial management included intravenous (IV) 0.9% normal saline (1000 mL every 24 hours), omeprazole (40 mg IV every 24 hours diluted in 100 mL of saline), and acetylcysteine (300 mg IV every 8 hours). Given the patient’s baseline neurological condition and signs of respiratory deterioration, he was admitted to the Intensive Care Unit (ICU). ICU management included prophylactic anticoagulation with enoxaparin (Clexane) 40 mg subcutaneously every 24 hours and a stat dose of magnesium sulfate 20% (4 g IV). As seen in Table 1, laboratory results revealed leukopenia (3.7/mm^3^), thrombocytopenia (70/mm^3^), elevated mean corpuscular volume (103.3 fL), and elevated C-reactive protein (64 mg/L).

**Table 1:** Main biochemical results of examen carried out.

Exams	Jan 26	Jan 27	Jan 28	Jan 29	Reference value
Blood count					
White blood cells	3.7	3.7	4.2	4.7	5000-10,000/mm^3^
Lymphocytes	31.4	39.3	35.8	31.9	15-50%
Neutrophils	61.4	52.9	53.1	55.1	50-70%
Red blood cells	4.18	4.0	4.35	4.25	4.0-5.5 million/mm^3^
Hemoglobin	13.9	13.7	13.5	14.6	12-16 g/dL
Hematocrit	43.1	40.2	40.7	42.5	37-50%
Mean corpuscular volume	103.3	93.5	99.5	100.1	87.0-97.0 fL
Mean corpuscular hemoglobin	33.2	31.2	34.5	34.4	27-33 pg/cell
Platelet count	70	68	60	64	150,000-450,000/mm^3^
Glycemia	127	75.3	76.5	-	70-110 mg/dL
Electrolytes					
Calcium	8.0	8.3	8.4	9.4	8.5-10.5 mg/dL
Chloride	102.7	105.5	105.9	-	85-115 mEq/L
Magnesium	1.74	1.88	1.94	-	1.60-2.50 mg/dL
Phosphorus	3.38	3.88	4.1	-	2.50-5.0 mg/dL
Potassium	4.15	4.06	3.7	-	3.5-5.1 mEq/L
Sodium	141	144	141	-	135-155 mEq/L
Lipid profile					
Total cholesterol	142	-	-	-	160-200 mg/dL
Triglycerides	102	-	-	-	50-150 mg/dL
High density lipoprotein	42.5	-	-	-	0-65 mg/dL
Low density lipoprotein	79.5	-	-	-	0-150 mg/dL
Very low-density lipoprotein	20	-	-	-	35-160 mg/dL
Renal function					
Creatinine, serum	1.06	1.0	0.94	-	0.70-1.40 mg/dL
Blood urea nitrogen	25	15.10	26.8	-	15.0-50.0 mg/dL
Liver function					
Aspartate aminotransferase	22.2	-	-	-	0.0-40.0 U/L
Alanine aminotransferase	26.2	-	-	-	0.0-31.0 U/L
Albumin	4.3	3.9	3.9	3.9	3.5-5.0 g/dL
Nutritional profile					
Vitamin D	-	-	48.8	-	30-100 ng/mL
Vitamin B12	-	-	135	-	141-489 pg/mL
Folic acid	-	-	6.79	-	2.34-17.59 ng/mL
Iron studies					
Iron, serum	-	-	163	-	65-175 µg/dL
Ferritin	-	-	546.44	-	70-475 ng/mL
Metabolic profile					
Lactate dehydrogenase	-	-	360	-	230-460 U/L
Ammonia	-	-	-	98.0	18-72 µmol/L
Inflammatory markers					
Erythrocyte sedimentation rate	4.32	-	5	-	0-15 mm/h
C-reactive protein	64	-	78	96.6	0.0-6.0 mg/L
Procalcitonin	-	-	0.20	-	0.0-0.50 ng/mL
Infectious profile					
Dengue IgM	-	-	Negative	-	
Dengue IgG	-	-	Negative	-	
Therapeutic drug monitoring					
Carbamazepine, serum	-	5.4	-	-	4.0-10.0 µg/mL
Valproic acid, serum	-	-	-	73.3	50-100 µg/mL

**Figure 1:**
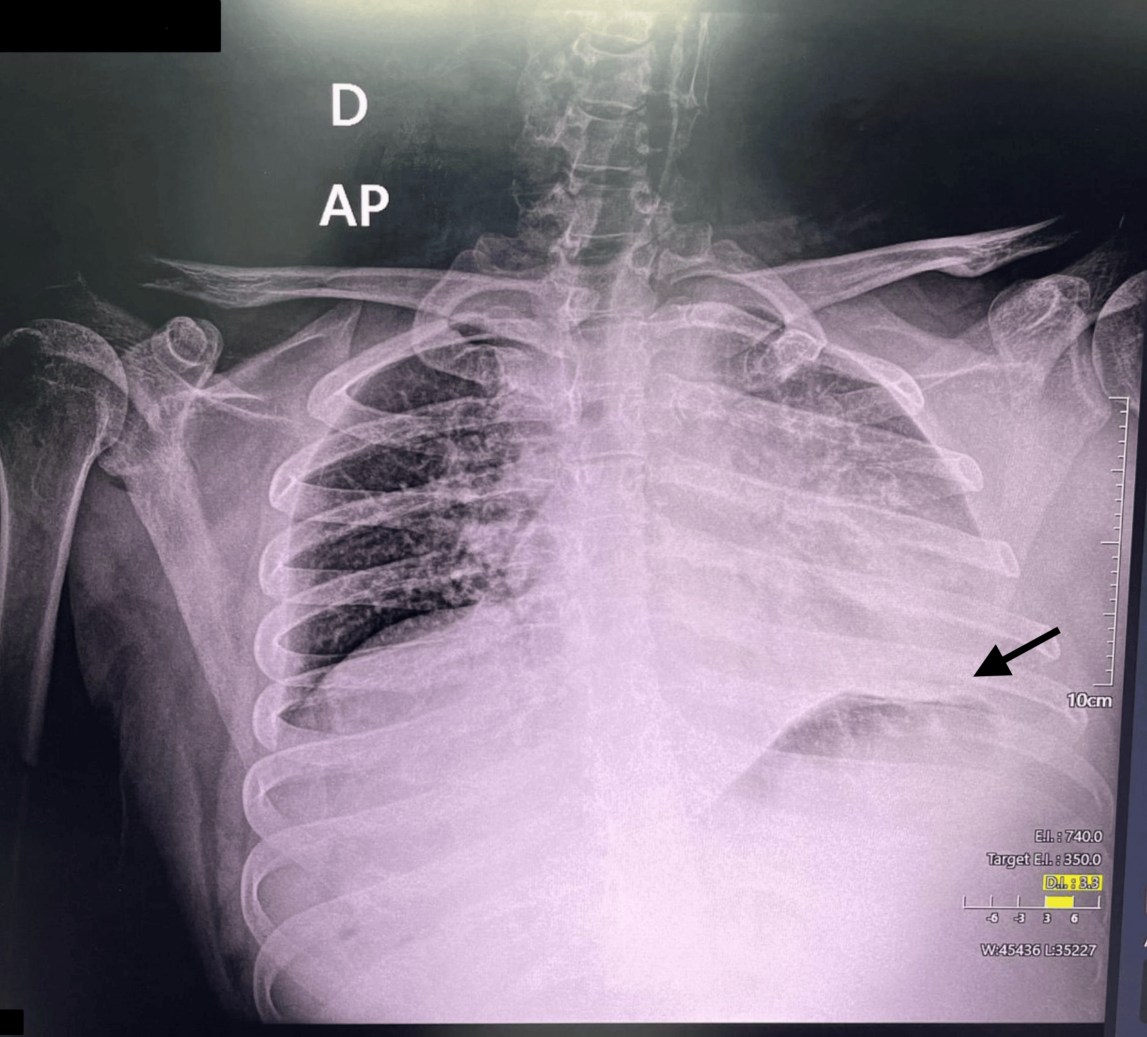
Initial chest X-ray showing a left basal-predominant interstitial infiltrate. AP: anteroposterior; D: derecho (right).

A chest X-ray showed a left basal-predominant interstitial infiltrate (Figure 1). Non-contrast cranial computed tomography (CT) revealed cerebral atrophy and no evidence of acute or chronic intracranial abnormalities. 

**Figure 2:**
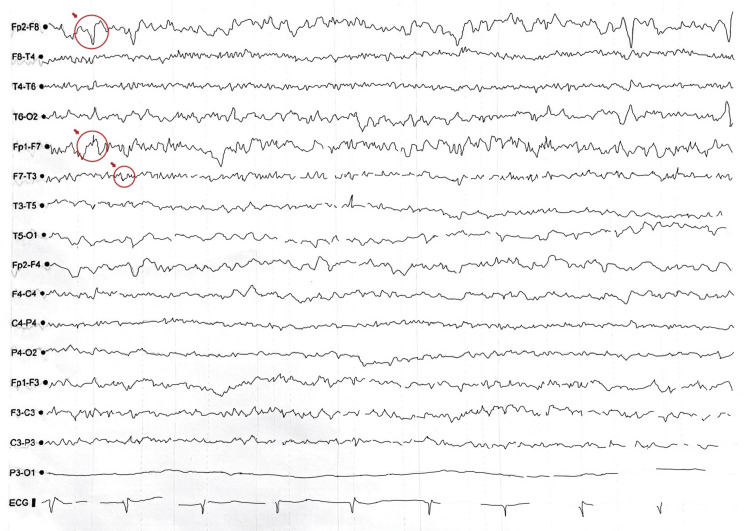
EEG showing frequent sharp waves with phase reversal observed in the left frontotemporal region. EEG: electroencephalogram.

Electroencephalogram (EEG) showed a baseline theta rhythm at 7-8 Hz with amplitudes of 20-50 µV. The tracing was symmetric and synchronous, with physiological reactivity to hyperventilation and intermittent photic stimulation. Notably, frequent sharp waves with phase reversal were observed in the left frontotemporal region. These findings were interpreted as an abnormal awake EEG consistent with cortical irritability (Figure 2).

On January 27, 2025, the patient remained somnolent but easily arousable. He appeared clinically hydrated and was on nasal cannula oxygen at 3 liters per minute. A multidisciplinary plan, in collaboration with the ICU team, included close monitoring, initiation of antimicrobial therapy, measurement of serum carbamazepine levels, and suspension of anticoagulation due to new-onset thrombocytopenia. The updated treatment regimen included magnesium sulfate 20% (10 mL diluted in 100 mL of 0.9% normal saline, as needed), acetylcysteine 300 mg IV every 8 hours, Ceftriaxone 1 g IV every 12 hours, metronidazole 500 mg IV every 8 hours, normal saline 1000 mL IV every 24 hours, and omeprazole 40 mg IV every 24 hours. 

Neurological reassessment described the patient as awake, though with a persistent tendency toward somnolence. He was afebrile, mildly confused, and in slight respiratory distress. He responded to painful stimuli and remained calm but appeared generally ill. The adjusted antiepileptic regimen included brivaracetam 100 mg orally every 12 hours, divalproex 500 mg orally every 12 hours, and clobazam 10 mg (2.5 tablets) orally at night. Follow-up labs showed leukopenia (3.7/mm^3^), thrombocytopenia (68/mm^3^), and serum carbamazepine levels within the reference range (Table 1). Carotid Doppler ultrasound showed no vascular pathology. Echocardiogram revealed normal cardiac anatomy and function, with a preserved left ventricular ejection fraction of 68%.

On January 28, 2025, the patient remained clinically stable, afebrile, and well hydrated. Supplemental oxygen continued via nasal cannula. ICU transfer to a general ward was considered based on improvement. The multidisciplinary plan included continued antibiotics and consultations with pulmonology and hematology. Additional labs were ordered: C-reactive protein, dengue IgM/IgG serology, and procalcitonin. The neurology department described him as clinically stable with no new neurological events. He was responsive and conversational, though occasionally incoherent. The team approved ward transfer and continuation of antiepileptic therapy.

Multidisciplinary management with pulmonology led to the following recommendations: continue antibiotics and add bronchodilators and corticosteroids, such as albuterol/ipratropium (1 ampoule as needed) and budesonide (0.75 mg inhaled every 12 hours). The hematologist found thrombocytopenia and macrocytosis raising suspicion for drug-induced cytopenia, likely from carbamazepine. A comprehensive workup was initiated (vitamin B12, folic acid, vitamin D, LDH, ESR, ferritin, serum iron).

The internal medicine assessment revealed that the patient was awake, mildly confused, somnolent, and in slight respiratory distress. Additional treatments included magnesium sulfate 20% (2 g IV every 24 hours), oral vitamin C (1 g daily), calcium gluconate 10% (1 ampoule IV every 12 hours diluted in 100 mL saline), and supportive fluids as needed. Laboratory findings reported: leukopenia (4.2/mm^3^, trending toward normal), MCV (99.5 fL), thrombocytopenia (60/mm^3^), low vitamin B12 levels (135 pg/mL), high ferritin (546.44 ng/mL), and CRP (78 mg/L).

**Figure 3:**
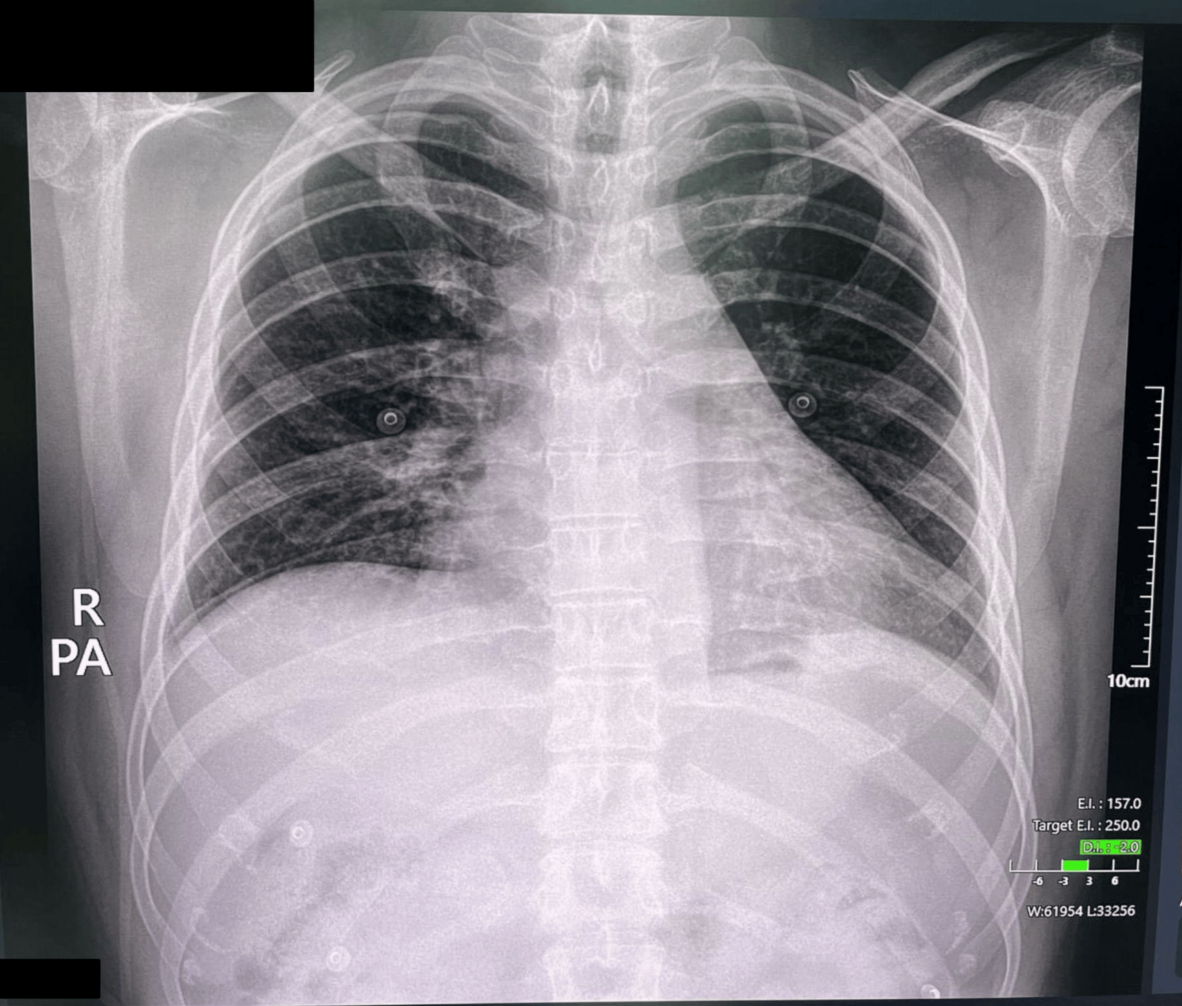
Follow-up chest X-ray. PA: posteroanterior; R: right.

By January 29, 2025, the patient was afebrile and eupneic with only a mild residual cough. A follow-up chest X-ray showed marked improvement (Figure 3). The pulmonologist deemed the patient stable for discharge. Final inpatient treatments included metronidazole 500 mg IV, ceftriaxone 1 g IV, acetylcysteine 300 mg IV, and inhaled albuterol/ipratropium and budesonide. 

A reevaluation by the hematology department through a peripheral smear revealed adequate mature white blood cells (4.3/mm^3^), normochromic red blood cells with a combination of normocytosis and macrocytosis, as well as the presence of ovalocytes and rouleaux formation. Mature lymphocytes were observed, and the platelet count was decreased (88/mm^3^), with some platelets exhibiting anisocytosis. These findings were consistent with megaloblastic changes suggestive of vitamin B12 deficiency. The ammonia result was received on the day of discharge, showing a slight elevation (98 µmol/L) without associated clinical symptoms. Outpatient follow-up was arranged.

The neurology team confirmed improved alertness and overall stabilization. With no seizures and consistent recovery, discharge was endorsed. The pulmonologist team confirmed resolution of the bronchial inflammation. The hematology department recommended outpatient follow-up for vitamin B12 deficiency, likely contributing to the macrocytosis and thrombocytopenia. The Internal medicine team endorsed the patient and recommended follow-up with the neurology department. 

The patient’s mother was thoroughly counseled on the importance of resuming the full antiepileptic regimen, including felbamate, which is currently unavailable in the Dominican Republic. Given the probable diagnosis of LGS and the high risk of status epilepticus, urgent return to the United States was advised to ensure access to his complete treatment protocol. The patient’s overall clinical evolution was favorable.

## Discussion

This case illustrates the potentially severe clinical consequences of substituting ASMs in patients with refractory epilepsy, particularly in those with suspected LGS, a condition known for its pharmacoresistance and the need for complex, individualized treatment regimens [7,8]. The patient, who had remained clinically stable for years on a tailored polypharmacologic regimen including felbamate, experienced a cascade of neurological and systemic complications following the inadvertent substitution of felbamate with carbamazepine. Some experts believed that carbamazepine should be used with caution due to the potential risk of aggravating drop seizures with a myoclonic component [9] and associated idiosyncratic hematologic reactions [10].

The clinical deterioration observed highlights the dangers of therapeutic interchangeability in this population, especially when medication changes are made without full awareness of epilepsy syndromic profiles and their pharmacologic contraindications. In LGS, inappropriate ASM selection can lead not only to seizure exacerbation [4] but also to hematological disturbances that may arise due to various mechanisms [11]. That is why it is crucial to select or develop an ASM that effectively controls seizure-related symptoms while minimizing adverse effects, particularly hematological side effects [11].

Felbamate is a second-generation agent reserved for severe epilepsies like LGS, but it carries a black box warning for aplastic anemia (which appears to be an age-dependent adverse effect) and hepatic failure [12]. However, in one randomized control trial [13], felbamate was initially recognized for its effectiveness in managing seizures associated with LGS when used as an add-on therapy. It demonstrated a notable reduction in the frequency of atonic and tonic seizures, along with improvements in quality of life. Subsequent long-term follow-up over a 12-month open-label period confirmed that these benefits were sustained [14]. Carbamazepine, in contrast, is contraindicated due to its potential to worsen absence, tonic, and myoclonic seizures, hallmark features of LGS, and its capacity to produce bone marrow suppression [4,15,16]. In this case, the replacement of felbamate with carbamazepine not only failed to control seizures but may have directly precipitated central nervous system depression and hematologic toxicity, likely due to additive effects in the context of a previously undiagnosed vitamin B12 deficiency.

The patient’s hematologic abnormalities appear to have been multifactorial. While vitamin B12 deficiency is itself associated with macrocytic anemia and thrombocytopenia, the progression and severity of these findings after ASM substitution suggest a compounding effect. Carbamazepine has been associated with both dose-dependent and idiosyncratic hematologic adverse events, including aplastic anemia, leukopenia, and thrombocytopenia [10]. In a vulnerable metabolic environment, such as B12 deficiency, the myelosuppressive effects of carbamazepine may be amplified, precipitating rapid decompensation [17]. 

Despite normal serum carbamazepine levels, the patient’s rapid clinical decline and hematologic abnormalities point toward carbamazepine toxicity. This paradox suggests that carbamazepine-induced adverse effects can manifest independently of elevated drug concentrations, particularly in the setting of metabolic vulnerabilities such as vitamin B12 deficiency. Vitamin B12 deficiency impairs DNA synthesis and hematopoiesis, potentially lowering the threshold for carbamazepine’s known myelosuppressive effects [10]. Moreover, carbamazepine’s metabolism may generate reactive intermediates capable of triggering idiosyncratic immune reactions affecting the bone marrow [11]. This case underscores the critical importance of preserving the integrity of individualized pharmacologic regimens in refractory epilepsy and avoiding substitutions without comprehensive clinical oversight. Baseline screening for vitamin and micronutrient deficiencies may be warranted prior to initiating or modifying high-risk ASMs, especially in patients with complex epilepsy syndromes and long-term polypharmacy.

## Conclusions

Beyond the pharmacologic implications, this case highlights systemic challenges in the management of chronic neurological conditions across borders. The lack of felbamate availability in the Dominican Republic, combined with caregiver misinterpretation, reflects a critical gap in medication continuity and health education. It is essential that discharge plans for internationally mobile patients include clear guidance, medication sourcing strategies, and liaison with providers in the host country.
